# Hepatic Mitochondria-Gut Microbiota Interactions in Metabolism-Associated Fatty Liver Disease

**DOI:** 10.3390/metabo13030322

**Published:** 2023-02-21

**Authors:** Francesco Bellanti, Aurelio Lo Buglio, Gianluigi Vendemiale

**Affiliations:** Department of Medical and Surgical Sciences, University of Foggia, 71122 Foggia, Italy

**Keywords:** liver mitochondria, gut microbiota, metabolism-associated fatty liver disease

## Abstract

The prevalence of metabolism-associated fatty liver disease (MAFLD) represents an urgent pandemic, complicated by a higher risk of morbidity and mortality as well as an increased socio-economic burden. There is growing evidence proving the impact of gut microbiota modifications on the development and progression of MAFLD through changes in metabolic pathways, modulation of the immune response, and activation of pro-inflammatory signals. Concurrently, metabolites produced by gut microbiota consisting of short chain fatty acids and bile acids contribute to the regulation of hepatic homeostasis by interacting with mitochondria. Evolving research indicates that innovative therapeutic targets for MAFLD may focus on gut microbiota–mitochondria interplay to regulate hepatic homeostasis. Recent investigations have explored the potential of new treatment strategies, such as prebiotics, probiotics, and metabolites, to change the composition of gut microbiota and simultaneously exert a positive impact on mitochondrial function to improve MAFLD. This review summarizes the significance of mitochondria and reports modifications in the composition of gut microbiota and its metabolites in MAFLD in order to illustrate the fascinating interplay between liver mitochondria and intestinal microbiota, discussing the potential effects of innovative treatments to modulate gut microbiota.

## 1. Introduction

Metabolic dysfunction-associated fatty liver disease (MAFLD), formerly named non-alcoholic fatty liver disease (NAFLD), is defined by the presence of steatosis in >5% of hepatic parenchyma associated with metabolic disorders (such as obesity and/or type 2 diabetes), in the absence of chronic conditions and with alcohol consumption lower than 30 g/d for men and 20 g/d for women [1]. MAFLD affects about a quarter of the world’s adult population, posing a major health and economic burden to all societies. However, despite this, it has no approved therapy [1,2,3,4]. The high prevalence of this disease has been fueled by the rapid rise in levels of sedentary behavior, low levels of physical activity, and excess calorie intake relative to expenditure in nutritionally imbalanced and unhealthy diets. In this context, the lack of effective prevention and treatment represents an urgent, unmet need in the field.

According to a recent International Consensus Panel, MAFLD can be diagnosed by liver biopsy, imaging, or blood biomarker evidence of fat accumulation in the liver (hepatic steatosis), in addition to one of the following three criteria, namely (1) overweight/ obesity, (2) the presence of type 2 diabetes mellitus (T2DM), or (3) evidence of metabolic dysregulation [1]. Liver fat accumulation is a very sensitive and early indicator of metabolic dysfunction [5,6]. Risk factors of MAFLD include advanced age, male gender, obesity, high levels of circulating metabolic parameters (such as fasting glucose, triglycerides, cholesterol, uric acid), urban residence, and lower education [7,8,9]. Hepatic steatosis implies a higher risk for atherosclerosis, and the relationship between MAFLD and atherosclerotic cardiovascular disease is sustained by huge epidemiological studies, genetic analyses, and investigations of subclinical atherosclerosis [10]. MAFLD may directly account for atherosclerotic cardiovascular disease by promoting hepatic delivery of atherogenic lipoproteins, particularly boosting secretion of very low-density lipoproteins [10]. Accordingly, cardiovascular disease is the most common cause of mortality in patients with fatty liver disease [11]. Furthermore, patients with MAFLD are exposed to higher risk of overall mortality, but also of more advanced liver disease and renal disease, worsening the prognosis of other diseases [12,13,14].

Ultrastructural and functional alterations of mitochondria are key determinants in the pathogenesis of MAFLD [15,16,17,18]. Being the powerhouse of cells, modifications of mitochondrial structure together with a decrease in mitochondrial function cause metabolic disorders and contribute to the progression of MAFLD [19,20,21]. Nevertheless, the exact mechanisms that link mitochondrial changes to steps of MAFLD progression need to be clarified.

The liver is the organ with the closest contact with the gut, since 75% of its blood supply comes from the portal vein, which collects blood from the intestine. Portal blood not only contains metabolites but also bacterial products and viable bacteria absorbed from the gut [22]. MAFLD is associated with an altered microbiota, and dysbiosis can increase toxic metabolites, cause liver inflammation and damage, aggravate liver disease, promote disease progression to liver fibrosis, and seriously affect patient outcomes [23]. On the other hand, liver alterations may exert an impact on intestinal microbiota. For example, bile transport from the liver to the small intestine through the biliary system is continuous, so hepatic changes resulting in a disruption to biliary homeostasis may have a negative effect on gut microbiota [24].

Mitochondria and gut microbiota closely interact, and a disruption of this interaction is reported in several conditions [25,26,27,28]. Indeed, intestinal microbiota and related metabolites are determinant for mitochondrial metabolism, biogenesis, and redox homeostasis [25]. On this basis, it is strongly conceivable that crosstalk between mitochondria and gut microbiota is crucial for the progression of MAFLD. In this review article, we will first summarize the importance of mitochondria in the pathogenesis of MAFLD. Next, changes in the composition of gut microbiota and its metabolites in MAFLD will be described. Finally, we will try to depict the intriguing interaction between liver mitochondria and intestinal microbiota, discussing the potential effects of innovative treatments to modulate gut microbiota in MAFLD.

## 2. Mitochondrial Involvement in MAFLD

In the liver, mitochondria represent almost one-fifth of the volume of hepatocytes (reaching a number of 500–4000 per cell), being involved in several key metabolic networks and signaling pathways [29]. One of the main mitochondrial functions is the production of ATP from energetic substrates as the products of carbohydrate, lipid and protein catabolism.

**Figure 1:**
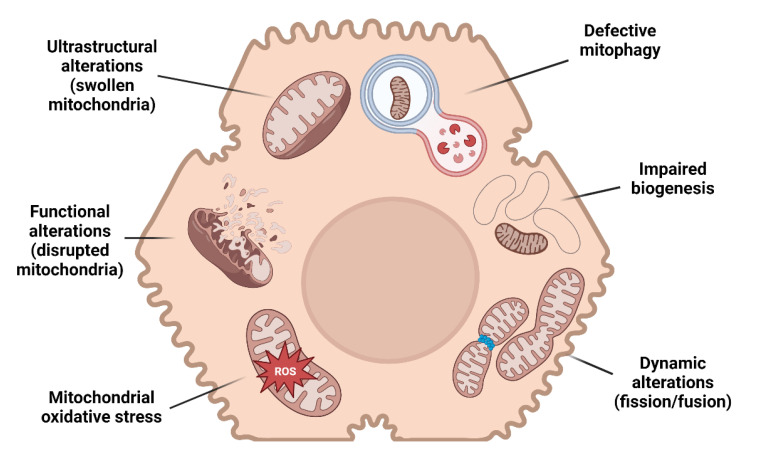
Mitochondrial alterations and MAFLD. A schematic illustration resuming the main mitochondrial changes in hepatocytes leading to MAFLD.

In the wide histological pattern characterizing MAFLD, simple steatosis supplemented by no or limited inflammation (non-alcoholic fatty liver) may progress to necro-inflammation with hepatocyte ballooning (non-alcoholic steatohepatitis, NASH) and fibrosis. Several studies provide evidence of mitochondrial alterations in every step of MAFLD progression (Figure 1).

Mitochondrial dysfunction is characterized by different grades of ultrastructural and morphologic modifications, impairment of β-oxidation, oxidative phosphorylation (OXPHOS) and ATP exhaustion, higher permeability of membranes, overproduction of reactive species, and oxidative damage of mitochondrial DNA (mtDNA) [30,31,32,33].

Mitochondrial morphology is commonly described as an elongated ovoid or spherical shape, depending on the plane and orientation of the sectioning of electron microscopy observation [34]. These organelles are enveloped by an outer and an inner membrane, from which parallel infoldings of cristae originate, functionally representing the surface area for ATP production [35,36]. The association between ultrastructural alterations and mitochondrial dysfunction is of particular interest because giant mitochondria characterize the occurrence of several diseases [37,38,39]. Of note, MAFLD patients present with ultrastructural changes consistent with giant mitochondria, characterized by a significant decrease in the surface area-to-volume ratio and disruption to the inner- and cristae mitochondrial membranes suggestive of mitochondrial dysfunction [40].

Liver steatosis develops as a consequence of increased lipid uptake and synthesis and/or reduced lipid removal/oxidation [19,20]. The lipid excess in liver steatosis is mainly composed of neutral lipids, such as triglycerides and cholesterol esters, which are gathered as lipid droplets in hepatocytes [41]. Hydrolysis of triglycerides and cholesterol esters releases fatty acids, which can be esterified again, incorporated in second messengers (such as phosphatidylinositol bisphosphate, sphingosine, ceramide, and others), or transported within mitochondria for β-oxidation [42]. Nevertheless, very-long-chain fatty acids cannot be oxidized by mitochondria, so these are transported to peroxisomes [43]. While medium and short-chain fatty acids can freely enter mitochondria, long-chain fatty acids need to be activated by CoA to be transported within the mitochondrial matrix through the carnitine palmitoyl transferase system [44]. Mitochondria are involved in fatty acid catabolism through β-oxidation reactions, whose impairment causes lipid accumulation in liver cells [45]. Reduced β-oxidation in MAFLD may be the consequence of decreased mitochondrial intake of fatty acids, mediated by the impaired activity of carnitine palmitoyl transferase 1 (CPT1) [46]. Indeed, CPT1 represents the mitochondrial gateway for long-chain fatty acid entrance within the matrix, being the key regulator of mitochondrial β-oxidation [47]. In mammals, CPT1 requires carnitine to translocate long-chain acyl CoAs across the inner mitochondrial membrane, and intramitochondrial carnitine reacts with short- and medium-chain acyl CoAs to generate short-chain acylcarnitine, which can be transported out of mitochondria [48]. The accumulation of long-chain acylcarnitine and reduction in short-chain acylcarnitine have been reported in the liver of MAFLD patients, further confirming the impaired mitochondrial β-oxidation of long-chain acyl-CoAs to medium- and short-chain acyl-CoAs [49]. On the other hand, it has been reported that the genetic deficiency in mitochondrial trifunctional protein (involved in the oxidation of long chain fatty acids) is associated with hepatic steatosis and insulin resistance [50,51]. Of note, mitochondrial fatty acid oxidation is decreased by 40–50% in patients with steatohepatitis, suggesting that the impairment of lipid metabolism is linked with disease progression [52].

Mitochondria also exert a determinant role in lipid synthesis. Indeed, even though lipogenesis occurs in the cytoplasm, the mitochondrial matrix is the producing site of citrate, the precursor of fatty acid synthesis [53]. Thus, lipid synthesis requires citrate export out of mitochondria, which occurs through a carrier reaction mediated by the ATP-dependent citrate lyase [54]. Of interest, ATP-citrate lyase is upregulated in patients with MAFLD, and its increased acetylation is associated with progression of disease [55].

In hepatic steatosis, hepatic mitochondrial β-oxidation is inefficient for the metabolism of fatty acid excess, so lipotoxic intermediates accumulate in the liver; furthermore, overstimulated mitochondrial β-oxidation generates excess reducing equivalents (NADH and FADH2) and electron flow to the electron transport chain (ETC), but this rate is limited by the ATP rate turnover, which impairs reactive species transfer and production [56].

MAFLD severity may be further influenced by the dysfunctional mitochondrial electron transport chain (ETC), with consequent OXPHOS defects and mitochondrial oxidative stress [57]. Impaired ETC is described in MAFLD patients, particularly in those presenting with steatohepatitis [49,58]. In particular, a human study reports that mitochondrial respiratory activity adapts during the progression of MAFLD, being increased with respect to mitochondrial mass in simple steatosis but compromised in steatohepatitis [58]. ETC defects and related overproduction of reactive species may lead to overexpression of uncoupling protein 2, with consequent bioenergetic impairment and ATP depletion [30,31]. Furthermore, MAFLD patients exhibit several mtDNA mutations involving ETC-related genes, and the number of mutations is associated with histologic severity [59].

Mitochondrial dysfunction is a consequence of altered mitochondrial quality control (MQC), which includes biogenesis, mitophagy and dynamics (fusion/fission) [60]. The transcription factor peroxisome proliferator-activated receptor γ (PPAR γ)-coactivator-1α (PGC-1α) is the master regulator of mitochondrial biogenesis and promotes the expression of genes engaged in β-oxidation, the tricarboxylic acid cycle, ETC, and mtDNA duplication [61]. The hepatic expression of PGC-1α is reduced in MAFLD patients [58]. Furthermore, PGC-1α and mitochondrial biogenesis are repressed in the liver of animal models of MAFLD [62,63]. Patients with MAFLD also exhibit alterations in mitochondrial protein synthesis [64]. Impairment of mitochondrial transcription factor A (TFAM), a downstream effector of PGC-1α that promotes mtDNA transcription and mtDNA repair enzymes, can lead to the disruption of mtDNA-encoded respiratory chain subunits, further amplifying mitochondrial dysfunction, and leading to the progression of steatohepatitis [63].

Providing the dysfunctional mitochondria is removed, mitophagy could be a protective mechanism to prevent MAFLD progression [65]. Mitophagy is defined as the cellular process that leads to the selective degradation of dysfunctional mitochondria, being the exclusive mechanism to specifically clear whole organelles and limit overproduction of reactive species [66]. As a result, defective mitophagy results in the accumulation of injured mitochondria, with consequent hepatocellular necrosis [67]. The main pathway regulating mitophagy and consequent mitochondrial function/structure is dependent on PINK1, which phosphorylates Parkin; in turn, activated Parkin mediates the ubiquitination of mitochondrial proteins, leading to the formation of an autophagosome and initiating the mitochondrial degradation program [68]. Pre-clinical evidence suggests that PINK1/Parkin mediated mitophagy is involved in hepatic lipid accumulation, suggesting its involvement in the pathogenesis of MAFLD [69,70]. Further pathways of mitophagy regulated by BCL2 interacting protein 3 (BNIP3)/BNIP3L (or NIX), FUN14 Domain Containing 1 (FUNDC1), AMP kinase (AMPK), mitochondria-eating protein (MIEAP), and high mobility group box 1 (HMGB1) may be involved in MAFLD pathogenesis [71].

Dysfunctional mitochondria can be further recovered through fusion with healthy organelles, even though severe impairment leads to segregation from mitochondrial network through fission [72]. Mitochondrial dynamics is modulated by several key proteins including mitochondrial fission 1 and 2 (Fis1 and Fis2), mitofusins (Mfn), mitochondrial fission factor (Mff), dynamin-related protein 1 (Drp1), and mitochondrial dynamic proteins of 49 kDa and 51 kDa (MiD49 and MiD51) [73]. Both Fis1 and Drp1 levels were shown to decrease in a mouse model characterized by liver inflammation and fibrosis [74]. On the other hand, hepatic expression of mitochondrial fission-related proteins such as Mff and Drp1 was found to be higher in mice fed HFD [75]. The importance of mitochondrial dynamics in MAFLD pathogenesis is strengthened by demonstrating that hepatic ablation of Mff increases the susceptibility of steatohepatitis development in HFD-fed mice [76]. Furthermore, hepatic Mfn2 levels are lower both in mouse models and in patients with steatosis/steatohepatitis [17]. Of note, Mfn2 re-expression is able to improve liver pathology in steatohepatitis, while the hepatic deletion of Mfn2 causes steatosis, inflammation, fibrosis, and liver cancer [77].

## 3. The Gut-Liver Axis: A Link between MAFLD and Intestinal Microbiota

Gut microbiota includes numerous microorganisms, such as bacteria, viruses, fungi, and archaea, within the gastrointestinal tract. Amongst the intestinal bacterial microbiota, *Firmicutes* and *Bacteroidetes* represent the prevalent phyla [78]. Gut microbiota exerts various determinant functions in preserving healthy homeostasis, avoiding colonization by pathogens, processing xenobiotics, and supplying vitamins, especially biotin and folate, which are implicated in energy metabolism and maintenance of the immune system [79].

**Figure 2:**
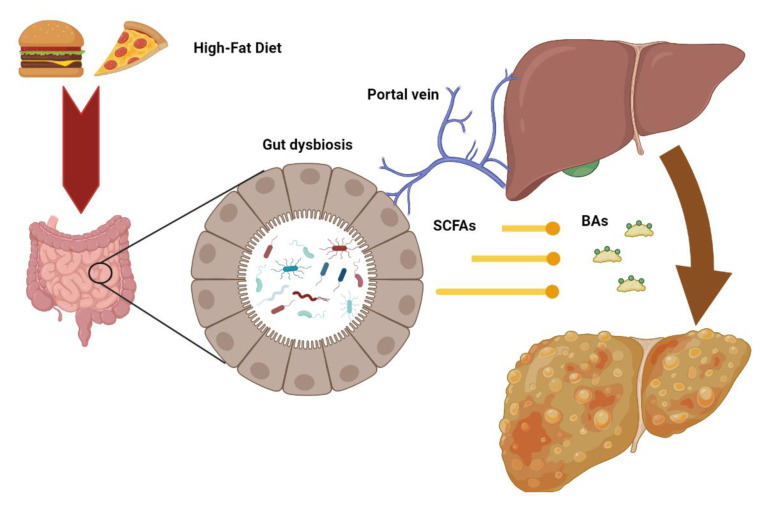
Intestinal dysbiosis consequent to metabolic alterations may increase the release of microbial metabolites such as short chain fatty acids (SCFD) and secondary bile acids (BAs) to the liver through the portal vein, contributing to the pathogenesis of hepatic injury.

Considering the bidirectional relationship between the liver and intestine, changes in gut microbiota—defined as “dysbiosis”—may contribute to several liver diseases, including steatosis, steatohepatitis, and fibrosis (Figure 2) [80].

MAFLD is promoted by nutritional changes, resulting in either overnutrition or malnutrition, so that the high-fat diet (HFD) closely mimics human liver disease in animals [81]. HFD reduces the Shannon diversity index and the special operational taxonomic units (OTUs) of gut microbiota, significantly increasing the wealth of *Firmicutes* and decreasing *Bacteroidetes* [82]. It is worth noting that similar changes in gut microbiota are described in humans with obesity [83]. Emerging evidence suggests that HFD plays a causal role in determining gut dysbiosis because of its increased ability for energy accumulation and higher gut inflammation and permeability [84]. On the other hand, the role of gut microbiota in the pathogenesis of MAFLD has been demonstrated by transplantation experiments in germ-free mice fed HFD: colonization of fecal microbiota from donors with hyperglycemia and high plasma levels of pro-inflammatory cytokines induces macrovesicular liver steatosis and increases the expression of genes involved in lipid synthesis [85]. It is worth noting that mice developing steatosis display a high concentration of two bacterial species (*Lachnospiraceae bacterium 609* and a relative of *Barnesiella intestinihominis*) [85]. Moreover, another investigation reports that hepatic steatosis may occur in wild type mice simply by co-housing mice with steatohepatitis due to inflammasome-mediated gut dysbiosis [86]. Nevertheless, information obtained by mouse models cannot be entirely transferred to humans, not only because hepatic histological alterations are not identical, but also because the microbiota is considerably dissimilar [87]. To sidestep this hindrance, fecal microbiota transplanted from MAFLD patients to germ-free mice to duplicate the human phenotype causes hepatic steatosis and inflammation, which are aggravated by HFD feeding [88].

The composition of gut microbiota in MAFLD patients has been investigated by a small number of studies, which led to heterogeneous results but described the presence of dysbiosis, confirming its contribution to the disease. In general, these studies suggest the reduction of gut microbiota diversity, with increased *Firmicutes* and *Actinomycetes* and reduced *Bacteroidetes*, *Leptosphaeria*, *Proteobacteria*, *Fusobacteria*, *Verrucomicrobia,* and *Thermus* [89,90,91]. Studies provide conflicting data for *Bacteroidetes* and *Actinobacteria*. It is also essential to point out that due to their close relationship, MAFLD and metabolic diseases such as obesity and type 2 diabetes may present with some overlap in bacterial signatures [92]. However, several differences are reported among investigations, with diverse results for phylum, family, genus, and species [93]. Despite the fact that most research on the gut-liver axis has focused on bacteria, patients with MAFLD have a unique fecal mycobiome composition and a strong systemic immune response to *Candida albicans* [94]. Furthermore, the diversity of the fecal virome was reduced in MAFLD patients compared to healthy subjects [95].

The profile of gut microbiota may be related to the severity of liver injury: its β-diversity is higher in cirrhosis than in the absence of advanced fibrosis [96]. Dysbiosis causes excess gut bacteria and increases lipid permeability and intake; furthermore, bacteria translocate to the portal circulation and trigger inflammatory signals via the detection of lipopolysaccharide, stimulating the expression of the toll-like receptor 4, production of IL-8, and inhibition of insulin signaling, ultimately increasing the influx of free fatty acids and lipotoxicity [97]. The latter is defined as the cytoplasmic accumulation of toxic lipids, causing organelle impairment, cellular injury, and apoptosis [98]. It is also worth noting that the diversity of both the fecal mycobiome and virome is associated with the severity of MAFLD [94,95].

Alteration of the gut microbiota may contribute to MAFLD pathogenesis through the products of microbial metabolism. Gut dysbiosis is associated with higher intestinal production of short chain fatty acids (SCFAs), which promote the transport of monosaccharides, gluconeogenesis, synthesis, and accumulation of toxic lipids in the liver [99]. Originating from carbohydrate fermentation by gut microbiota, acetic acid, propionic acid and butyric acid are the most representative intestinal SCFAs: acetic acid produces 10% of total daily energy for the host, while butyric acid is the main energy producer for intestinal epithelial cells [100]. Germ-free models and mice with antibiotic-induced microbiome depletion exhibit reduced turnover and decreased proliferative activity of intestinal epithelial barrier, with consequent increased gut permeability [101,102]. Furthermore, butyric acid may promote hepatic lipid synthesis and consequent lipotoxicity though its modulation of Carbohydrate Response Element Binding Protein (ChREBP) and Sterol Response Element Binding Protein-1 (SREBP-1), the main controllers of de novo lipogenesis [102]. Propionic acid is involved in MAFLD pathogenesis, acting as a direct precursor for lipogenesis [103]. A further mechanism through which SCFAs may induce hepatic lipotoxicity is related to their capacity to activate G protein-coupled receptors [104]. In particular, the activation of GPR43 triggers hepatic lipogenesis, stimulating the development of MAFLD [105].

A further cause of gut microbiota-induced lipotoxicity is represented by excess secondary bile acids (SBAs). SBAs are converted by intestinal microorganism from primary bile acids, which preserve gut microbiota homeostasis by directly inhibiting pathogenic bacteria and activating the farnesoid X receptor (FXR) [106]. The FXR contributes to the preservation of the intestinal epithelial barrier and down-regulates the expression of SREBP-1c and liver X receptor (LXR), decreasing hepatic lipogenesis [107].

Other gut microbiota-related metabolites, such as ethanol, choline, and tryptophan derivatives, may be implicated in MAFLD pathogenesis [108]. Ethanol derived from intestinal microbiota is considered to be a determinant for MAFLD pathogenesis, since its chronic microbial production may impair glucose and lipid metabolism, inducing hepatic steatosis and steatohepatitis [109]. Gut microbiota-derived ethanol levels in portal vein blood of patients with MAFLD are higher than healthy subjects, and correlate with increased fecal concentrations of lactic acid bacteria [110]. Choline and its derivatives, such as trimethylamine (TMA) and TMA-N-oxide (TMAO), are the main microbial metabolites, whose high amounts are associated with lipid metabolism and atherosclerosis [111]. Low choline levels may induce hepatic lipid accumulation by decreasing the synthesis and secretion of very low-density lipoproteins (VLDL) in humans, replicating that described in rodents fed a methionine/choline deficient (MCD) diet [112]. TMA translocates through the portal circulation to the liver, where it is transformed to TMAO, which promotes and aggravates liver steatosis [113,114]. Microbial derivatives of tryptophan, such as tryptamine and indole-3-acetate, are able to favorably modulate the hepatic inflammatory response, but HFD induces changes in gut microbiota associated with the depletion of tryptophan metabolites [115].

An integrative analysis of gut microbiota and fecal metabolites performed in MAFLD patients, in addition to describing a reduced variety of species, reported modifications in circulating and fecal microbial-derived metabolites with prevailing lipid molecules [116]. Thus, the complete characterization of the pathogenic role of gut microbiota in MAFLD development and progression must include the intricate interaction with microbial metabolites.

## 4. Interplay between Gut Microbiota and Liver Mitochondria: A New Perspective of Pathway Communication

There is a strong relationship between mitochondria and microbes, suggesting a possible interaction among these organelles and gut microbiota. Recent investigations describe a bidirectional relationship between mitochondria and gut microbiota [117]. Indeed, mitochondria evolved from within a bacterial phylum (either *Alphaproteobacteria* or an ancestor related to *Rickettsiales* were hypothesized), so that they share several proteins for parallel metabolic pathways [118,119,120]. Early mitochondria established a reliable symbiosis with ancient anaerobic bacteria, since they were capable of providing energy even in the absence of oxygen, while nutrients were provided by the host [121]. As the complexity of the organisms increased, new forms of symbiosis were required, so single-cell communication was expanded toward multicellularity to allow remote connections between the microbiota and the host cells, promoting composite metabolic pathways. Thus, resident mitochondria are now part of the host, and microbiota mainly supplies metabolites that are required by mitochondria to maintain host metabolism [121]. The strong connection between mitochondria and gut microbiota is further supported by the evidence that bactericidal antibiotics such as aminoglycosides, quinolones, and beta-lactam antibiotics may lead to mitochondrial dysfunction [122]. On the other hand, mitochondrial-targeted antioxidants may also be effective as antibiotics [123].

Variations in gut microbiota and related metabolites may promote the excessive production of mitochondrial reactive species, leading to the loss of redox homeostasis and oxidative damage, which are key pathogenetic factors for the progression of steatosis and the development of steatohepatitis in MAFLD [124,125]. Interplay between gut microbiota and mitochondria may be triggered by metabolites such as short chain fatty acids and bile acids, but also by direct modulation of gene expression [126]. Changes in gut microbiota induced by HFD decrease the available pool of short chain fatty acids, which are energetic substrates for mitochondria [127,128]. Furthermore, HFD triggers the conversion of conjugated bile acids to secondary bile acids— which are able to modulate mitochondrial function via transcription factors implicated in carbohydrate and lipid metabolism—through Bifidobacterium and Bacteroides [129]. Both animal and human MAFLD are associated with high circulating levels of TVAMA (a metabolite of gut microbiota), which in turn reduces carnitine levels, impairing the mitochondrial internalization and consequent β-oxidation of fatty acids and alters the preservation of mitochondrial structure [130].

The association of MAFLD pathophysiology, mitochondrial dysfunction and gut dysbiosis is suggested by studies that mostly used culture-independent methods, relying on metagenomic or DNA sequencing [93]. *Dysosmobacter welbionis*, a novel species of *Ruminococcaceae* isolated from feces of obese and diabetic patients, is negatively correlated with metabolic markers; furthermore, administration of *D. welbionis* to HFD-fed mice reduces fat mass by increasing lipolysis and mitochondrial number/function [131].

On the other hand, mitochondria may modulate gut microbiota. More than regulating the innate immune response against pathogen infection, mitochondria are involved in the control of the intestinal epithelial barrier: indeed, mitochondrial dysfunction facilitates transepithelial flux of *Escherichia coli* [132]. The analysis of gut microbiota in models of mitochondrial dysfunction demonstrates that variations in mitochondrial genetics change the composition of microbial community, which is associated with host production of reactive species [133].

Evidence of an interaction between gut microbiota and liver mitochondria is also provided by studies that approached MAFLD treatment with novel functional foods or fecal transplantation, targeting the microbiota but exerting an impact on mitochondrial function [126]. The prebiotic xylo-oligosaccharides (XOS)—which target *F. prausnitzii*—improves liver steatosis in HFD-fed rats, but it is also able to boost hepatic β-3 Hydroxyacyl Coenzyme A Dehydrogenase activity and complex I levels, and to improve oxidative phosphorylation and ATP generation [134,135]. Additionally, XOS increase lipid oxidation in the liver of patients with steatohepatitis through depletion of isovalerate and tyrosine [136]. The prebiotic resistant dextrin (indigestible dextrin) limits mitochondrial dysfunction and reduces the production of reactive species by favorable modulation of gut microbiota (boosting *Blautia* and *Dubosiella*, which increase the amount of lactitol, cafestol, dimethyl fumarate, and 4-hydroxyphenylacetic acid), ameliorating liver steatosis in HFD-fed mice [137,138]. The effects of resistant dextrin are also mediated by the reduction in secondary bile acids and tryptophan, and the increase in indole derivatives, which play a role as endogenous metabolites to decrease inflammatory response and intestinal permeability in MAFLD [139,140]. Active peptides such as Val-Val-Tyr-Pro (VVYP)—a component of the globin digest—may prevent steatohepatitis by re-establishing gut microbiota balance [141]. Indeed, VVYP may increase *Coriobacteriaceae*, *Eubacteriaceae*, *Bacteroidia*, and *Desulfovibrionaceae* to change the amount of short chain fatty acids and bile acids; this effect is associated with a reduction in the fragmentation of swollen and vacuolate mitochondria, improvement in lipid metabolism, and a reduction in circulating pro-inflammatory cytokines [141]. Finally, transplantation of cecal microbiota from methylation-controlled J protein (inhibitor of mitochondrial complex I activity) knockout to germ-free mice delayed steatohepatitis and ameliorated gut-liver axis in a forceful dietary model of advanced MAFLD [142].

Microbial metabolites may exert beneficial effects on MAFLD by improving mitochondrial function. The protective impact of SCFAs on MAFLD has been well demonstrated in both animal investigations and human studies; indeed, these fatty acids (1) modulate liver mitochondria energy metabolism by supplying reducing equivalents, and (2) decrease the production of mitochondrial reactive species [143]. Of note, administration of butyrate or acetate to different rodent models of MAFLD reduced liver steatosis and inflammation, while targeted delivery of propionate to colon decreased intrahepatic lipid concentration in overweight patients [144,145,146].

Interestingly, chronic exposure to HFD increases the catabolism of choline by intestinal *E. coli* through injured mitochondrial bioenergetics in colonic epithelial cells, which in turn raises concentration of potentially harmful choline derivative TMAO [147]. Whether this mechanism occurs also in liver mitochondria, or whether *E. coli*-derived TMAO is a main responsible for hepatic steatosis in MAFLD is not clear and is worth of investigation.

Interventions that act synergistically on gut microbiota and mitochondrial function may represent novel therapeutic strategies for the treatment of MAFLD. Increased intake of mono- (olive oil) and polyunsaturated fatty acids (soybean oil), rather than saturated fatty acids (coconut oil), increase gut microbiota diversity and symbiotic balance and improve fatty acid oxidation as well as mitochondrial respiration, increasing insulin sensitivity and preventing liver steatosis [148]. Berberine, an oral hypoglycemic plant extract with anti-obesity and hypolipidemic properties, is able to prevent dysbiosis induced by HFD, together with mitochondrial impairment observed in colon enterocytes [149]. Nevertheless, whether berberine could improve mitochondrial function in hepatocytes is worthy of investigation. The antioxidant resveratrol—a natural polyphenol—exhibits hepatoprotective effects associated with favorable changes in gut microbiota and the induction of antioxidant enzymes by triggering the Nuclear factor erythroid 2-related factor 2 (NRF2) [150]. Curcumin, another natural polyphenol with antioxidant properties, exerts beneficial effects in MAFLD not only by reducing oxidative stress and improving mitochondrial function, but also modulating gut microbiota and circulating microbial metabolites [151,152]. Even the natural polyphenol quercetine shows favorable effects in hepatic steatosis and steatohepatitis through positive modulation of mitochondrial fatty acids metabolism and redox homeostasis, but also through the induction of a protective phenotype of gut microbiota [153].

Overall, this evidence reinforces the significance of microbiota-mitochondria interplay in MAFLD, suggesting that focusing on it could be a helpful treatment strategy.

## 5. Conclusions

In the last few years, our knowledge of the intestinal system has been improved, particularly in regard to its relationship with liver function. The interplay between the liver and gut microbiota, in the context of the gut-liver axis, has been well described by several investigations. Most of these studies proved that the structure, mass, function, and dynamics of liver mitochondria can be modulated by the composition of gut microbiota. Furthermore, even though studies on the direct regulation of liver mitochondria by gut microbiota, or on the liver modulation of gut microbiota composition through mitochondrial signaling are scarce, this evidence had been progressively described in recent times. Considering the pivotal role of both gut microbiota and mitochondria in the pathogenesis of MAFLD, innovative therapeutics may target the interaction between these factors to improve hepatic homeostasis. This perspective paper suggests a theoretical model about the interplay between gut microbiota and liver mitochondria, relying on the common evolutionary homology between mitochondria and bacteria. Symbiont and pathobiont bacteria may control liver mitochondria. The interplay between liver mitochondria and intestinal microbiota across the portal circulation represents a major pathway of the liver-gut axis. This new perspective not only develops our knowledge of the liver-gut interaction, but also suggests a new therapeutic approach, targeting the liver mitochondria-gut microbiota relationship with the potential to treat MAFLD.

## References

[1] Eslam M., Sanyal A.J., George J. (2019). Toward More Accurate Nomenclature for Fatty Liver Diseases. Gastroenterology.

[2] Younossi Z.M. (2019). Non-alcoholic fatty liver disease—A global public health perspective. J. Hepatol..

[3] Sarin S.K., Kumar M., Eslam M., George J., Al Mahtab M., Akbar S.M.F., Jia J., Tian Q., Aggarwal R., Muljono D.H. (2019). Liver diseases in the Asia-Pacific region: A Lancet Gastroenterology & Hepatology Commission. Lancet Gastroenterol. Hepatol..

[4] Zupo R., Castellana F., Panza F., Castellana M., Lampignano L., Cincione R., Triggiani V., Giannelli G., Dibello V., Sardone R. (2021). Non Alcoholic Fatty Liver Disease Is Positively Associated with Increased Glycated Haemoglobin Levels in Subjects without Diabetes. J. Clin. Med..

[5] Bril F., Cusi K. (2017). Liver fat accumulation as a barometer of insulin responsiveness again points to adipose tissue as the culprit. Hepatology.

[6] Micek A., Godos J., Cernigliaro A., Cincione R.I., Buscemi S., Libra M., Galvano F., Grosso G. (2021). Total Nut, Tree Nut, and Peanut Consumption and Metabolic Status in Southern Italian Adults. Int. J. Environ. Res. Public Health.

[7] Chen Y.-L., Li H., Li S., Xu Z., Tian S., Wu J., Liang X.-Y., Li X., Liu Z.-L., Xiao J. (2021). Prevalence of and risk factors for metabolic associated fatty liver disease in an urban population in China: A cross-sectional comparative study. BMC Gastroenterol..

[8] Yuan Q., Wang H., Gao P., Chen W., Lv M., Bai S., Wu J. (2022). Prevalence and Risk Factors of Metabolic-Associated Fatty Liver Disease among 73,566 Individuals in Beijing, China. Int. J. Environ. Res. Public Health.

[9] Guan L., Zhang X., Tian H., Jin X., Fan H., Wang N., Sun J., Li D., Li J., Wang X. (2022). Prevalence and risk factors of metabolic-associated fatty liver disease during 2014–2018 from three cities of Liaoning Province: An epidemiological survey. BMJ Open.

[10] Stols-Goncalves D., Hovingh G.K., Nieuwdorp M., Holleboom A.G. (2019). NAFLD and Atherosclerosis: Two Sides of the Same Dysmetabolic Coin?. Trends Endocrinol. Metab..

[11] Tsutsumi T., Eslam M., Kawaguchi T., Yamamura S., Kawaguchi A., Nakano D., Koseki M., Yoshinaga S., Takahashi H., Anzai K. (2021). MAFLD better predicts the progression of atherosclerotic cardiovascular risk than NAFLD: Generalized estimating equation approach. Hepatol. Res..

[12] Yamamura S., Eslam M., Kawaguchi T., Tsutsumi T., Nakano D., Yoshinaga S., Takahashi H., Anzai K., George J., Torimura T. (2020). MAFLD identifies patients with significant hepatic fibrosis better than NAFLD. Liver Int..

[13] Sun D.-Q., Jin Y., Wang T.-Y., Zheng K.I., Rios R.S., Zhang H.-Y., Targher G., Byrne C.D., Yuan W.-J., Zheng M.-H. (2020). MAFLD and risk of CKD. Metabolism.

[14] Kim D., Konyn P., Sandhu K.K., Dennis B.B., Cheung A.C., Ahmed A. (2021). Metabolic dysfunction-associated fatty liver disease is associated with increased all-cause mortality in the United States. J. Hepatol..

[15] Cioffi F., Giacco A., Petito G., de Matteis R., Senese R., Lombardi A., de Lange P., Moreno M., Goglia F., Lanni A. (2022). Altered Mitochondrial Quality Control in Rats with Metabolic Dysfunction-Associated Fatty Liver Disease (MAFLD) Induced by High-Fat Feeding. Genes.

[16] Fahlbusch P., Nikolic A., Hartwig S., Jacob S., Kettel U., Köllmer C., Al-Hasani H., Lehr S., Müller-Wieland D., Knebel B. (2022). Adaptation of Oxidative Phosphorylation Machinery Compensates for Hepatic Lipotoxicity in Early Stages of MAFLD. Int. J. Mol. Sci..

[17] Ramanathan R., Ali A.H., Ibdah J.A. (2022). Mitochondrial Dysfunction Plays Central Role in Nonalcoholic Fatty Liver Disease. Int. J. Mol. Sci..

[18] Scalcon V., Folda A., Lupo M.G., Tonolo F., Pei N., Battisti I., Ferri N., Arrigoni G., Bindoli A., Holmgren A. (2022). Mitochondrial depletion of glutaredoxin 2 induces metabolic dysfunction-associated fatty liver disease in mice. Redox Biol..

[19] Serviddio G., Sastre J., Bellanti F., Viña J., Vendemiale G., Altomare E. (2008). Mitochondrial involvement in non-alcoholic steatohepatitis. Mol. Asp. Med..

[20] Serviddio G., Bellanti F., Vendemiale G., Altomare E. (2011). Mitochondrial dysfunction in nonalcoholic steatohepatitis. Expert Rev. Gastroenterol. Hepatol..

[21] Fromenty B., Roden M. (2022). Mitochondrial alterations in fatty liver diseases. J. Hepatol..

[22] Schatten W.E., Desprez J.D., Holden W.D. (1955). A Bacteriologic Study of Portal-Vein Blood in Man. Arch. Surg..

[23] Duarte S.M., Stefano J.T., Oliveira C.P. (2019). Microbiota and nonalcoholic fatty liver disease/nonalcoholic steatohepatitis (NAFLD/NASH). Ann. Hepatol..

[24] Lang S., Schnabl B. (2020). Microbiota and Fatty Liver Disease-the Known, the Unknown, and the Future. Cell Host Microbe.

[25] Huang Y., Xin W., Xiong J., Yao M., Zhang B., Zhao J. (2022). The Intestinal Microbiota and Metabolites in the Gut-Kidney-Heart Axis of Chronic Kidney Disease. Front. Pharmacol..

[26] Olubodun-Obadun T.G., Ishola I.O., Adeyemi O.O. (2022). Impact of environmental toxicants exposure on gut-brain axis in Parkinson disease. Drug Metab. Pers. Ther..

[27] Peng H., Yu S., Zhang Y., Yin Y., Zhou J. (2022). Intestinal Dopamine Receptor D2 is Required for Neuroprotection Against 1-Methyl-4-phenyl-1,2,3,6-tetrahydropyridine-induced Dopaminergic Neurodegeneration. Neurosci. Bull..

[28] Zhu Y., Li Y., Zhang Q., Song Y., Wang L., Zhu Z. (2022). Interactions Between Intestinal Microbiota and Neural Mitochondria: A New Perspective on Communicating Pathway from Gut to Brain. Front. Microbiol..

[29] Degli Esposti D., Hamelin J., Bosselut N., Saffroy R., Sebagh M., Pommier A., Martel C., Lemoine A. (2012). Mitochondrial Roles and Cytoprotection in Chronic Liver Injury. Biochem. Res. Int..

[30] Serviddio G., Bellanti F., Tamborra R., Rollo T., Romano A.D., Giudetti A.M., Capitanio N., Petrella A., Vendemiale G., Altomare E. (2008). Alterations of hepatic ATP homeostasis and respiratory chain during development of non-alcoholic steatohepatitis in a rodent model. Eur. J. Clin. Investig..

[31] Serviddio G., Bellanti F., Tamborra R., Rollo T., Capitanio N., Romano A.D., Sastre J., Vendemiale G., Altomare E. (2008). Uncoupling protein-2 (UCP2) induces mitochondrial proton leak and increases susceptibility of non-alcoholic steatohepatitis (NASH) liver to ischaemia-reperfusion injury. Gut.

[32] Bellanti F., Villani R., Tamborra R., Blonda M., Iannelli G., di Bello G., Facciorusso A., Poli G., Iuliano L., Avolio C. (2017). Synergistic interaction of fatty acids and oxysterols impairs mitochondrial function and limits liver adaptation during nafld progression. Redox Biol..

[33] Li Z., Li Y., Zhang H., Guo J., Lam C.W.K., Wang C., Zhang W. (2019). Mitochondria-Mediated Pathogenesis and Therapeutics for Non-Alcoholic Fatty Liver Disease. Mol. Nutr. Food Res..

[34] Palade G.E. (1952). The fine structure of mitochondria. Anat. Rec..

[35] Perkins G., Renken C., Martone M., Young S., Ellisman M., Frey T. (1997). Electron Tomography of Neuronal Mitochondria: Three-Dimensional Structure and Organization of Cristae and Membrane Contacts. J. Struct. Biol..

[36] Frey T.G., Mannella C.A. (2000). The internal structure of mitochondria. Trends Biochem. Sci..

[37] Cataldo A.M., McPhie D.L., Lange N.T., Punzell S., Elmiligy S., Ye N.Z., Froimowitz M.P., Hassinger L.C., Menesale E.B., Sargent L.W. (2010). Abnormalities in Mitochondrial Structure in Cells from Patients with Bipolar Disorder. Am. J. Pathol..

[38] Mannella C.A., Lederer W.J., Jafri M.S. (2013). The connection between inner membrane topology and mitochondrial function. J. Mol. Cell. Cardiol..

[39] Cogliati S., Frezza C., Soriano M.E., Varanita T., Quintana-Cabrera R., Corrado M., Cipolat S., Costa V., Casarin A., Gomes L.C. (2013). Mitochondrial Cristae Shape Determines Respiratory Chain Supercomplexes Assembly and Respiratory Efficiency. Cell.

[40] Shami G.J., Cheng D., Verhaegh P., Koek G., Wisse E., Braet F. (2021). Three-dimensional ultrastructure of giant mitochondria in human non-alcoholic fatty liver disease. Sci. Rep..

[41] Gluchowski N.L., Becuwe M., Walther T.C., Farese R.V. (2017). Lipid droplets and liver disease: From basic biology to clinical implications. Nat. Rev. Gastroenterol. Hepatol..

[42] Dabravolski S., Bezsonov E., Baig M., Popkova T., Orekhov A. (2021). Mitochondrial Lipid Homeostasis at the Crossroads of Liver and Heart Diseases. Int. J. Mol. Sci..

[43] Chornyi S., Ijlst L., Van Roermund C.W.T., Wanders R.J.A., Waterham H.R. (2021). Peroxisomal Metabolite and Cofactor Transport in Humans. Front. Cell Dev. Biol..

[44] Miotto P.M., Petrick H.L., Holloway G.P. (2020). Acute insulin deprivation results in altered mitochondrial substrate sensitivity conducive to greater fatty acid transport. Am. J. Physiol. Metab..

[45] Ucar F., Sezer S., Erdogan S., Akyol S., Armutcu F., Akyol O. (2013). The relationship between oxidative stress and nonalcoholic fatty liver disease: Its effects on the development of nonalcoholic steatohepatitis. Redox Rep..

[46] Serviddio G., Giudetti A.M., Bellanti F., Priore P., Rollo T., Tamborra R., Siculella L., Vendemiale G., Altomare E., Gnoni G.V. (2011). Oxidation of Hepatic Carnitine Palmitoyl Transferase-I (CPT-I) Impairs Fatty Acid Beta-Oxidation in Rats Fed a Methionine-Choline Deficient Diet. PLoS ONE.

[47] Eaton S. (2002). Control of mitochondrial beta-oxidation flux. Prog. Lipid Res..

[48] Bremer J. (1983). Carnitine--metabolism and functions. Physiol. Rev..

[49] Perez-Carreras M., Del H.P., Martin M.A., Rubio J.C., Martin A., Castellano G., Colina F., Arenas J., Solis-Herruzo J.A. (2003). Defective hepatic mitochondrial respiratory chain in patients with nonalcoholic steatohepatitis. Hepatology.

[50] Ibdah J.A., Perlegas P., Zhao Y., Angdisen J., Borgerink H., Shadoan M.K., Wagner J.D., Matern D., Rinaldo P., Cline J.M. (2005). Mice Heterozygous for a Defect in Mitochondrial Trifunctional Protein Develop Hepatic Steatosis and Insulin Resistance. Gastroenterology.

[51] Rector R.S., Morris E.M., Ridenhour S., Meers G.M., Hsu F.-F., Turk J., Ibdah J.A. (2013). Selective hepatic insulin resistance in a murine model heterozygous for a mitochondrial trifunctional protein defect. Hepatology.

[52] Moore M.P., Cunningham R.P., Meers G.M., Johnson S.A., Wheeler A.A., Ganga R.R., Spencer N.M., Pitt J.B., Diaz-Arias A., Swi A.I.A. (2022). Compromised hepatic mitochondrial fatty acid oxidation and reduced markers of mitochondrial turnover in human NAFLD. Hepatology.

[53] Pizzuto R., Paventi G., Atlante A., Passarella S. (2010). Pyruvate kinase in pig liver mitochondria. Arch. Biochem. Biophys..

[54] Chen Z., Yu Y., Cai J., Li H. (2019). Emerging Molecular Targets for Treatment of Nonalcoholic Fatty Liver Disease. Trends Endocrinol. Metab..

[55] Guo L., Guo Y.-Y., Li B.-Y., Peng W.-Q., Chang X.-X., Gao X., Tang Q.-Q. (2019). Enhanced acetylation of ATP-citrate lyase promotes the progression of nonalcoholic fatty liver disease. J. Biol. Chem..

[56] Di Ciaula A., Passarella S., Shanmugam H., Noviello M., Bonfrate L., Wang D.Q., Portincasa P. (2021). Nonalcoholic Fatty Liver Disease (NAFLD). Mitochondria as Players and Targets of Therapies?. Int. J. Mol. Sci..

[57] Reinecke F., Smeitink J.A., van der Westhuizen F.H. (2009). OXPHOS gene expression and control in mitochondrial disorders. Biochim. Biophys. Acta (BBA)-Mol. Basis Dis..

[58] Koliaki C., Szendroedi J., Kaul K., Jelenik T., Nowotny P., Jankowiak F., Herder C., Carstensen M., Krausch M., Knoefel W.T. (2015). Adaptation of Hepatic Mitochondrial Function in Humans with Non-Alcoholic Fatty Liver Is Lost in Steatohepatitis. Cell Metab..

[59] Sookoian S., Flichman D., Scian R., Rohr C., Dopazo H., Gianotti T.F., Martino J.S., Castaño G.O., Pirola C.J. (2016). Mitochondrial genome architecture in non-alcoholic fatty liver disease. J. Pathol..

[60] Sheldon R., Meers G.M., Morris E.M., Linden M., Cunningham R.P., Ibdah J.A., Thyfault J.P., Laughlin M.H., Rector R.S. (2019). eNOS deletion impairs mitochondrial quality control and exacerbates Western diet-induced NASH. Am. J. Physiol. Metab..

[61] Halling J.F., Pilegaard H. (2020). PGC-1α-mediated regulation of mitochondrial function and physiological implications. Appl. Physiol. Nutr. Metab..

[62] Nadal-Casellas A., Amengual-Cladera E., Proenza A.M., Lladó I., Gianotti M. (2010). Long-term High-fat-diet Feeding Impairs Mitochondrial Biogenesis in Liver of Male and Female Rats. Cell. Physiol. Biochem..

[63] Aharoni-Simon M., Hann-Obercyger M., Pen S., Madar Z., Tirosh O. (2011). Fatty liver is associated with impaired activity of PPARgamma-coactivator 1alpha (PGC1alpha) and mitochondrial biogenesis in mice. Lab. Investig..

[64] Mansouri A., Gattolliat C.-H., Asselah T. (2018). Mitochondrial Dysfunction and Signaling in Chronic Liver Diseases. Gastroenterology.

[65] Madrigal-Matute J., Cuervo A.M. (2016). Regulation of Liver Metabolism by Autophagy. Gastroenterology.

[66] Kurihara Y., Kanki T., Aoki Y., Hirota Y., Saigusa T., Uchiumi T., Kang D. (2012). Mitophagy Plays an Essential Role in Reducing Mitochondrial Production of Reactive Oxygen Species and Mutation of Mitochondrial DNA by Maintaining Mitochondrial Quantity and Quality in Yeast. J. Biol. Chem..

[67] Ashrafi G., Schwarz T.L. (2012). The pathways of mitophagy for quality control and clearance of mitochondria. Cell Death Differ..

[68] Urbina-Varela R., Castillo N., Videla L.A., Del Campo A. (2020). Impact of Mitophagy and Mitochondrial Unfolded Protein Response as New Adaptive Mechanisms Underlying Old Pathologies: Sarcopenia and Non-Alcoholic Fatty Liver Disease. Int. J. Mol. Sci..

[69] Liu P., Lin H., Xu Y., Zhou F., Wang J., Liu J., Zhu X., Guo X., Tang Y., Yao P. (2018). Frataxin-Mediated PINK1-Parkin-Dependent Mitophagy in Hepatic Steatosis: The Protective Effects of Quercetin. Mol. Nutr. Food Res..

[70] Chen D., Ran D., Wang C., Liu Y., Ma Y., Song R., Gao Y., Liu Z. (2021). Role of mitochondrial dysfunction and PINK1/Parkin-mediated mitophagy in Cd-induced hepatic lipid accumulation in chicken embryos. Life Sci..

[71] Zhu L., Wu X., Liao R. (2023). Mechanism and regulation of mitophagy in nonalcoholic fatty liver disease (NAFLD): A mini-review. Life Sci..

[72] Li R., Toan S., Zhou H. (2020). Role of mitochondrial quality control in the pathogenesis of nonalcoholic fatty liver disease. Aging.

[73] Wang B., Xiao X., Huang F., Liu R. (2019). Syntaxin-17-Dependent Mitochondrial Dynamics Is Essential for Protection against Oxidative-Stress-Induced Apoptosis. Antioxidants.

[74] Krishnasamy Y., Gooz M., Li L., Lemasters J.J., Zhong Z. (2019). Role of mitochondrial depolarization and disrupted mitochondrial homeostasis in non-alcoholic steatohepatitis and fibrosis in mice. Int. J. Physiol. Pathophysiol. Pharmacol..

[75] Galloway C.A., Lee H., Brookes P., Yoon Y. (2014). Decreasing mitochondrial fission alleviates hepatic steatosis in a murine model of nonalcoholic fatty liver disease. Am. J. Physiol. Liver Physiol..

[76] Takeichi Y., Miyazawa T., Sakamoto S., Hanada Y., Wang L., Gotoh K., Uchida K., Katsuhara S., Sakamoto R., Ishihara T. (2021). Non-alcoholic fatty liver disease in mice with hepatocyte-specific deletion of mitochondrial fission factor. Diabetologia.

[77] Hernández-Alvarez M.I., Sebastián D., Vives S., Ivanova S., Bartoccioni P., Kakimoto P., Plana N., Veiga S.R., Hernández V., Vasconcelos N. (2019). Deficient Endoplasmic Reticulum-Mitochondrial Phosphatidylserine Transfer Causes Liver Disease. Cell.

[78] Rinninella E., Raoul P., Cintoni M., Franceschi F., Miggiano G.A.D., Gasbarrini A., Mele M.C. (2019). What Is the Healthy Gut Microbiota Composition? A Changing Ecosystem across Age, Environment, Diet, and Diseases. Microorganisms.

[79] Vezza T., Abad-Jiménez Z., Marti-Cabrera M., Rocha M., Víctor V.M. (2020). Microbiota-Mitochondria Inter-Talk: A Potential Therapeutic Strategy in Obesity and Type 2 Diabetes. Antioxidants.

[80] Khan A., Ding Z., Ishaq M., Bacha A.S., Khan I., Hanif A., Li W., Guo X. (2021). Understanding the Effects of Gut Microbiota Dysbiosis on Nonalcoholic Fatty Liver Disease and the Possible Probiotics Role: Recent Updates. Int. J. Biol. Sci..

[81] Im Y.R., Hunter H., Hahn D.D.G., Duret A., Cheah Q., Dong J., Fairey M., Hjalmarsson C., Li A., Lim H.K. (2021). A Systematic Review of Animal Models of NAFLD Finds High-Fat, High-Fructose Diets Most Closely Resemble Human NAFLD. Hepatology.

[82] Ding Q., Guo R., Pei L., Lai S., Li J., Yin Y., Xu T., Yang W., Song Q., Han Q. (2022). *N*-Acetylcysteine alleviates high fat diet-induced hepatic steatosis and liver injury *via* regulating the intestinal microecology in mice. Food Funct..

[83] Magne F., Gotteland M., Gauthier L., Zazueta A., Pesoa S., Navarrete P., Balamurugan R. (2020). The Firmicutes/Bacteroidetes Ratio: A Relevant Marker of Gut Dysbiosis in Obese Patients?. Nutrients.

[84] Murphy E.A., Velazquez K.T., Herbert K.M. (2015). Influence of high-fat diet on gut microbiota: A driving force for chronic disease risk. Curr. Opin. Clin. Nutr. Metab. Care.

[85] Le Roy T., Llopis M., Lepage P., Bruneau A., Rabot S., Bevilacqua C., Martin P., Philippe C., Walker F., Bado A. (2012). Intestinal microbiota determines development of non-alcoholic fatty liver disease in mice. Gut.

[86] Henao-Mejia J., Elinav E., Jin C., Hao L., Mehal W.Z., Strowig T., Thaiss C.A., Kau A.L., Eisenbarth S.C., Jurczak M.J. (2012). Inflammasome-mediated dysbiosis regulates progression of NAFLD and obesity. Nature.

[87] Nguyen T.L., Vieira-Silva S., Liston A., Raes J. (2015). How informative is the mouse for human gut microbiota research?. Dis. Model. Mech..

[88] Chiu C.-C., Ching Y.-H., Li Y.-P., Liu J.-Y., Huang Y.-T., Huang Y.-W., Yang S.-S., Huang W.-C., Chuang H.-L. (2017). Nonalcoholic Fatty Liver Disease Is Exacerbated in High-Fat Diet-Fed Gnotobiotic Mice by Colonization with the Gut Microbiota from Patients with Nonalcoholic Steatohepatitis. Nutrients.

[89] Boursier J., Mueller O., Barret M., Machado M., Fizanne L., Araujo-Perez F., Guy C.D., Seed P.C., Rawls J.F., David L.A. (2016). The severity of nonalcoholic fatty liver disease is associated with gut dysbiosis and shift in the metabolic function of the gut microbiota. Hepatology.

[90] Gómez-Zorita S., Aguirre L., Milton-Laskibar I., Fernández-Quintela A., Trepiana J., Kajarabille N., Mosqueda-Solís A., González M., Portillo M.P. (2019). Relationship between Changes in Microbiota and Liver Steatosis Induced by High-Fat Feeding—A Review of Rodent Models. Nutrients.

[91] Pan X., Wen S.W., Kaminga A.C., Liu A. (2020). Gut metabolites and inflammation factors in non-alcoholic fatty liver disease: A systematic review and meta-analysis. Sci. Rep..

[92] Hernández-Ceballos W., Cordova-Gallardo J., Mendez-Sanchez N. (2021). Gut Microbiota in Metabolic-associated Fatty Liver Disease and in Other Chronic Metabolic Diseases. J. Clin. Transl. Hepatol..

[93] Aron-Wisnewsky J., Vigliotti C., Witjes J., Le P., Holleboom A.G., Verheij J., Nieuwdorp M., Clément K. (2020). Gut microbiota and human NAFLD: Disentangling microbial signatures from metabolic disorders. Nat. Rev. Gastroenterol. Hepatol..

[94] Demir M., Lang S., Hartmann P., Duan Y., Martin A., Miyamoto Y., Bondareva M., Zhang X., Wang Y., Kasper P. (2021). The fecal mycobiome in non-alcoholic fatty liver disease. J. Hepatol..

[95] Lang S., Demir M., Martin A., Jiang L., Zhang X., Duan Y., Gao B., Wisplinghoff H., Kasper P., Roderburg C. (2020). Intestinal Virome Signature Associated with Severity of Nonalcoholic Fatty Liver Disease. Gastroenterology.

[96] Caussy C., Tripathi A., Humphrey G., Bassirian S., Singh S., Faulkner C., Bettencourt R., Rizo E., Richards L., Xu Z.Z. (2019). A gut microbiome signature for cirrhosis due to nonalcoholic fatty liver disease. Nat. Commun..

[97] Woldeamlak B., Yirdaw K., Biadgo B. (2019). Role of Gut Microbiota in Type 2 Diabetes Mellitus and Its Complications: Novel Insights and Potential Intervention Strategies. Korean J. Gastroenterol..

[98] Marra F., Svegliati-Baroni G. (2018). Lipotoxicity and the gut-liver axis in NASH pathogenesis. J. Hepatol..

[99] Den Besten G., van Eunen K., Groen A.K., Venema K., Reijngoud D.-J., Bakker B.M. (2013). The role of short-chain fatty acids in the interplay between diet, gut microbiota, and host energy metabolism. J. Lipid Res..

[100] Zhou D., Chen Y.-W., Zhao Z.-H., Yang R.-X., Xin F.-Z., Liu X.-L., Pan Q., Zhou H., Fan J.-G. (2018). Sodium butyrate reduces high-fat diet-induced non-alcoholic steatohepatitis through upregulation of hepatic GLP-1R expression. Exp. Mol. Med..

[101] Slezak K., Krupova Z., Rabot S., Loh G., Levenez F., Descamps A., Lepage P., Doré J., Bellier S., Blaut M. (2014). Association of germ-free mice with a simplified human intestinal microbiota results in a shortened intestine. Gut Microbes.

[102] Park J.-H., Kotani T., Konno T., Setiawan J., Kitamura Y., Imada S., Usui Y., Hatano N., Shinohara M., Saito Y. (2016). Promotion of intestinal epithelial cell turnover by commensal bacteria: Role of short-chain fatty acids. PLoS ONE.

[103] Liu W., Luo X., Tang J., Mo Q., Zhong H., Zhang H., Feng F. (2020). A bridge for short-chain fatty acids to affect inflammatory bowel disease, type 1 diabetes, and non-alcoholic fatty liver disease positively: By changing gut barrier. Eur. J. Nutr..

[104] Lu Y., Fan C., Li P., Lu Y., Chang X., Qi K. (2016). Short Chain Fatty Acids Prevent High-fat-diet-induced Obesity in Mice by Regulating G Protein-coupled Receptors and Gut Microbiota. Sci. Rep..

[105] Kimura T., Pydi S.P., Pham J., Tanaka N. (2020). Metabolic Functions of G Protein-Coupled Receptors in Hepatocytes—Potential Applications for Diabetes and NAFLD. Biomolecules.

[106] Fiorucci S., Distrutti E. (2019). The Pharmacology of Bile Acids and Their Receptors. Bile Acids Recept..

[107] Han X., Cui Z.-Y., Song J., Piao H.-Q., Lian L.-H., Hou L.-S., Wang G., Zheng S., Dong X.-X., Nan J.-X. (2019). Acanthoic acid modulates lipogenesis in nonalcoholic fatty liver disease via FXR/LXRs-dependent manner. Chem. Interactions.

[108] Vallianou N., Christodoulatos G.S., Karampela I., Tsilingiris D., Magkos F., Stratigou T., Kounatidis D., Dalamaga M. (2021). Understanding the Role of the Gut Microbiome and Microbial Metabolites in Non-Alcoholic Fatty Liver Disease: Current Evidence and Perspectives. Biomolecules.

[109] Jeon S., Carr R. (2020). Alcohol effects on hepatic lipid metabolism. J. Lipid Res..

[110] Meijnikman A.S., Davids M., Herrema H., Aydin O., Tremaroli V., Rios-Morales M., Levels H., Bruin S., de Brauw M., Verheij J. (2022). Microbiome-derived ethanol in nonalcoholic fatty liver disease. Nat. Med..

[111] Li X., Su C., Jiang Z., Yang Y., Zhang Y., Yang M., Zhang X., Du Y., Zhang J., Wang L. (2021). Berberine attenuates choline-induced atherosclerosis by inhibiting trimethylamine and trimethylamine-N-oxide production via manipulating the gut microbiome. Npj Biofilms Microbiomes.

[112] Zhang J., Zhao Y., Wang S., Li G., Xu K. (2022). CREBH alleviates mitochondrial oxidative stress through SIRT3 mediating deacetylation of MnSOD and suppression of Nlrp3 inflammasome in NASH. Free. Radic. Biol. Med..

[113] Tan X., Liu Y., Long J., Chen S., Liao G., Wu S., Li C., Wang L., Ling W., Zhu H. (2019). Trimethylamine *N* -Oxide Aggravates Liver Steatosis through Modulation of Bile Acid Metabolism and Inhibition of Farnesoid X Receptor Signaling in Nonalcoholic Fatty Liver Disease. Mol. Nutr. Food Res..

[114] Shi C., Pei M., Wang Y., Chen Q., Cao P., Zhang L., Guo J., Deng W., Wang L., Li X. (2022). Changes of flavin-containing monooxygenases and trimethylamine-N-oxide may be involved in the promotion of non-alcoholic fatty liver disease by intestinal microbiota metabolite trimethylamine. Biochem. Biophys. Res. Commun..

[115] Krishnan S., Ding Y., Saedi N., Choi M., Sridharan G.V., Sherr D.H., Yarmush M.L., Alaniz R.C., Jayaraman A., Lee K. (2018). Gut Microbiota-Derived Tryptophan Metabolites Modulate Inflammatory Response in Hepatocytes and Macrophages. Cell Rep..

[116] Yang L., Dai Y., He H., Liu Z., Liao S., Zhang Y., Liao G., An Z. (2022). Integrative analysis of gut microbiota and fecal metabolites in metabolic associated fatty liver disease patients. Front. Microbiol..

[117] Clark A., Mach N. (2017). The Crosstalk between the Gut Microbiota and Mitochondria during Exercise. Front. Physiol..

[118] Karlberg O., Canback B., Kurland C.G., Andersson S.G. (2000). The dual origin of the yeast mitochondrial proteome. Yeast.

[119] Wang Z., Wu M. (2015). An integrated phylogenomic approach toward pinpointing the origin of mitochondria. Sci. Rep..

[120] Fan L., Wu D., Goremykin V., Xiao J., Xu Y., Garg S., Zhang C., Martin W.F., Zhu R. (2020). Phylogenetic analyses with systematic taxon sampling show that mitochondria branch within Alphaproteobacteria. Nat. Ecol. Evol..

[121] Franco-Obregón A., Gilbert J.A. (2017). The Microbiome-Mitochondrion Connection: Common Ancestries, Common Mechanisms, Common Goals. Msystems.

[122] Kalghatgi S., Spina C.S., Costello J.C., Liesa M., Morones-Ramirez J.R., Slomovic S., Molina A., Shirihai O.S., Collins J.J. (2013). Bactericidal Antibiotics Induce Mitochondrial Dysfunction and Oxidative Damage in Mammalian Cells. Sci. Transl. Med..

[123] Nazarov P.A., Osterman I.A., Tokarchuk A.V., Karakozova M.V., Korshunova G.A., Lyamzaev K.G., Skulachev M.V., Kotova E.A., Skulachev V.P., Antonenko Y.N. (2017). Mitochondria-targeted antioxidants as highly effective antibiotics. Sci. Rep..

[124] Mossad O., Batut B., Yilmaz B., Dokalis N., Mezö C., Nent E., Nabavi L.S., Mayer M., Maron F.J.M., Buescher J.M. (2022). Gut microbiota drives age-related oxidative stress and mitochondrial damage in microglia via the metabolite N6-carboxymethyllysine. Nat. Neurosci..

[125] Shandilya S., Kumar S., Jha N.K., Kesari K.K., Ruokolainen J. (2021). Interplay of gut microbiota and oxidative stress: Perspective on neurodegeneration and neuroprotection. J. Adv. Res..

[126] Zhang Q., Xing W., Wang Q., Tang Z., Wang Y., Gao W. (2022). Gut microbiota–mitochondrial inter-talk in non-alcoholic fatty liver disease. Front. Nutr..

[127] Lumeng L., Davis E.J. (1973). The oxidation of acetate by liver mitochondria. FEBS Lett..

[128] Yan J., Xue Q., Chen W., Wang K., Peng D., Jiang J., Li P., Du B. (2022). Probiotic-fermented rice buckwheat alleviates high-fat diet-induced hyperlipidemia in mice by suppressing lipid accumulation and modulating gut microbiota. Food Res. Int..

[129] Jia W., Xie G., Jia W. (2018). Bile acid-microbiota crosstalk in gastrointestinal inflammation and carcinogenesis. Nat. Rev. Gastroenterol. Hepatol..

[130] Zhao M., Zhao L., Xiong X., He Y., Huang W., Liu Z., Ji L., Pan B., Guo X., Wang L. (2020). TMAVA, a Metabolite of Intestinal Microbes, Is Increased in Plasma from Patients With Liver Steatosis, Inhibits γ-Butyrobetaine Hydroxylase, and Exacerbates Fatty Liver in Mice. Gastroenterology.

[131] Le Roy T., de Hase E.M., Van H.M., Paquot A., Pelicaen R., Regnier M., Depommier C., Druart C., Everard A., Maiter D. (2022). Dysosmobacter welbionis is a newly isolated human commensal bacterium preventing diet-induced obesity and metabolic disorders in mice. Gut.

[132] Wang A., Keita V., Phan V., McKay C.M., Schoultz I., Lee J., Murphy M.P., Fernando M., Ronaghan N., Balce D. (2014). Targeting Mitochondria-Derived Reactive Oxygen Species to Reduce Epithelial Barrier Dysfunction and Colitis. Am. J. Pathol..

[133] Yardeni T., Tanes C.E., Bittinger K., Mattei L.M., Schaefer P.M., Singh L.N., Wu G.D., Murdock D.G., Wallace D.C. (2019). Host mitochondria influence gut microbiome diversity: A role for ROS. Sci. Signal..

[134] Gulhane M., Murray L., Lourie R., Tong H., Sheng Y.H., Wang R., Kang A., Schreiber V., Wong K.Y., Magor G. (2016). High Fat Diets Induce Colonic Epithelial Cell Stress and Inflammation that is Reversed by IL-22. Sci. Rep..

[135] Lensu S., Pariyani R., Mäkinen E., Yang B., Saleem W., Munukka E., Lehti M., Driuchina A., Lindén J., Tiirola M. (2020). Prebiotic Xylo-Oligosaccharides Ameliorate High-Fat-Diet-Induced Hepatic Steatosis in Rats. Nutrients.

[136] Gitto S., Schepis F., Andreone P., Villa E. (2018). Study of the Serum Metabolomic Profile in Nonalcoholic Fatty Liver Disease: Research and Clinical Perspectives. Metabolites.

[137] Zhao H., Jiang Z., Chang X., Xue H., Yahefu W., Zhang X. (2018). 4-Hydroxyphenylacetic Acid Prevents Acute APAP-Induced Liver Injury by Increasing Phase II and Antioxidant Enzymes in Mice. Front. Pharmacol..

[138] Dwivedi D.K., Jena G., Kumar V. (2020). Dimethyl fumarate protects thioacetamide-induced liver damage in rats: Studies on Nrf2, NLRP3, and NF-kappaB. J. Biochem. Mol. Toxicol..

[139] Park S.M., Lee J.R., Ku S.K., Cho I.J., Byun S.H., Kim S.C., Park S.J., Kim Y.W. (2015). Isoliquiritigenin in licorice functions as a hepatic protectant by induction of antioxidant genes through extracellular signal-regulated kinase-mediated NF-E2-related factor-2 signaling pathway. Eur. J. Nutr..

[140] Wlodarska M., Luo C., Kolde R., D’Hennezel E., Annand J.W., Heim C.E., Krastel P., Schmitt E.K., Omar A.S., Creasey E.A. (2017). Indoleacrylic Acid Produced by Commensal Peptostreptococcus Species Suppresses Inflammation. Cell Host Microbe.

[141] Xie X., Zhang L., Yuan S., Li H., Zheng C., Xie S., Sun Y., Zhang C., Wang R., Jin Y. (2021). Val-Val-Tyr-Pro protects against non-alcoholic steatohepatitis in mice by modulating the gut microbiota and gut-liver axis activation. J. Cell. Mol. Med..

[142] Juárez-Fernández M., Goikoetxea-Usandizaga N., Porras D., García-Mediavilla M.V., Bravo M., Serrano-Maciá M., Simón J., Delgado T.C., Lachiondo-Ortega S., Martínez-Flórez S. (2022). Enhanced mitochondrial activity reshapes a gut microbiota profile that delays NASH progression. Hepatology.

[143] Schonfeld P., Wojtczak L. (2016). Short- and medium-chain fatty acids in energy metabolism: The cellular perspective. J. Lipid Res..

[144] Jin C.J., Sellmann C., Engstler A.J., Ziegenhardt D., Bergheim I. (2015). Supplementation of sodium butyrate protects mice from the development of non-alcoholic steatohepatitis (NASH). Br. J. Nutr..

[145] Chambers E.S., Viardot A., Psichas A., Morrison D.J., Murphy K.G., Zac-Varghese S.E.K., MacDougall K., Preston T., Tedford C., Finlayson G.S. (2015). Effects of targeted delivery of propionate to the human colon on appetite regulation, body weight maintenance and adiposity in overweight adults. Gut.

[146] Dangana E., Omolekulo T., Areola E., Olaniyi K., Soladoye A., Olatunji L. (2019). Sodium acetate protects against nicotine-induced excess hepatic lipid in male rats by suppressing xanthine oxidase activity. Chem. Interactions.

[147] Yoo W., Zieba J.K., Foegeding N.J., Torres T.P., Shelton C.D., Shealy N.G., Byndloss A.J., Cevallos S.A., Gertz E., Tiffany C.R. (2021). High-fat diet–induced colonocyte dysfunction escalates microbiota-derived trimethylamine *N* -oxide. Science.

[148] López-Salazar V., Tapia M.S., Tobón-Cornejo S., Díaz D., Alemán-Escondrillas G., Granados-Portillo O., Noriega L., Tovar A.R., Torres N. (2021). Consumption of soybean or olive oil at recommended concentrations increased the intestinal microbiota diversity and insulin sensitivity and prevented fatty liver compared to the effects of coconut oil. J. Nutr. Biochem..

[149] Sun Y., Jin C., Zhang X., Jia W., Le J., Ye J. (2018). Restoration of GLP-1 secretion by Berberine is associated with protection of colon enterocytes from mitochondrial overheating in diet-induced obese mice. Nutr. Diabetes.

[150] Liao J.-X., Chen Y.-W., Shih M.-K., Tain Y.-L., Yeh Y.-T., Chiu M.-H., Chang S.K.C., Hou C.-Y. (2021). Resveratrol Butyrate Esters Inhibit BPA-Induced Liver Damage in Male Offspring Rats by Modulating Antioxidant Capacity and Gut Microbiota. Int. J. Mol. Sci..

[151] Chashmniam S., Mirhafez S.R., Dehabeh M., Hariri M., Nezhad M.A., Nobakht M.G.B. (2019). A pilot study of the effect of phospholipid curcumin on serum metabolomic profile in patients with non-alcoholic fatty liver disease: A randomized, double-blind, placebo-controlled trial. Eur. J. Clin. Nutr..

[152] Du S., Zhu X., Zhou N., Zheng W., Zhou W., Li X. (2022). Curcumin alleviates hepatic steatosis by improving mitochondrial function in postnatal overfed rats and fatty L02 cells through the SIRT3 pathway. Food Funct..

[153] Chen L., Liu J., Mei G., Chen H., Peng S., Zhao Y., Yao P., Tang Y. (2021). Quercetin and non-alcoholic fatty liver disease: A review based on experimental data and bioinformatic analysis. Food Chem. Toxicol..

